# *NSG2* (*ORF19.273*) Encoding Protein Controls Sensitivity of *Candida albicans* to Azoles through Regulating the Synthesis of C14-Methylated Sterols

**DOI:** 10.3389/fmicb.2018.00218

**Published:** 2018-02-20

**Authors:** Quan-Zhen Lv, Yu-Lin Qin, Lan Yan, Liang Wang, Chuyue Zhang, Yuan-Ying Jiang

**Affiliations:** ^1^Center for New Drug Research, College of Pharmacy, Second Military Medical University, Shanghai, China; ^2^Shanghai Pinghe Bilingual School, Shanghai, China

**Keywords:** *Candida albicans*, sterols, drug resistance, azoles, *NSG2*

## Abstract

Antifungal azole drugs inhibit the synthesis of ergosterol and cause the accumulation of sterols containing a 14α-methyl group, which is related to the properties of cell membrane. Due to the frequent recurrence of fungal infections and clinical long-term prophylaxis, azole resistance is increasing rapidly. In our research, Nsg2p, encoded by the *ORF19.273* in *Candida albicans*, is found to be involved in the inhibition of 14α-methylated sterols and resistance to azoles. Under the action of fluconazole, *nsg2*Δ/Δ mutants are seriously damaged in the integrity and functions of cell membranes with a decrease of ergosterol ratio and an increase of both obtusifoliol and 14α-methylfecosterol ratio. The balance between ergosterol and 14α-methyl sterols mediated by *NSG2* plays an important role in *C. albicans* responding to azoles *in vitro* as well as *in vivo*. These phenotypes are completely different from those of Nsg2p in *Saccharomyces cerevisia*e, which is proved to increase the stability of HMG-CoA and resistance to lovastatin. Based on the evidence above, it is indicated that the decrease of 14α-methylated sterols is an azole-resistant mechanism in *C. albicans*, which may provide new strategies for overcoming the problems of azole resistance.

## Introduction

*Candida albicans* is the major opportunistic pathogen that causes superficial infections as well as candidemia and deep-tissue infections in immunocompromised individuals (Kullberg and Arendrup, 2015). Approximately, 60% of all Candida infections are caused by *C. albicans* (Bassetti et al., 2013). Currently azoles, which target to the rate-limiting lanosterol demethylase (Erg11p) step in ergosterol biosynthesis, are the most prevalent antifungal drugs. However, azole-resistance induced by the prophylactic and prolonged treatment has become a serious problem in the clinic (Parker et al., 2014). In the Centers for Disease Control and Prevention reports published in 2013, azole-resistant *Candida* has been cited as a serious threat to human health. As a result, elucidating the resistance mechanisms of *C. albicans* and solving the problems of clinical azole-resistance has become a great challenge.

There are three classes of antifungal drugs targeted to the ergosterol synthetic pathway: allylamines, triazoles, and morpholines. Among these, triazoles have the lowest number of side effects. The antifungal mechanisms of triazoles include two aspects: one is the reduction of ergosterol and the other is the accumulation of aberrantly formed sterols with a C14 α-methyl group. These effects lead to damage of cell membranes (Lupetti et al., 2002; Martel et al., 2010a). In the fungal biomembrane system, ergosterol plays an indispensable role in coordinating membrane heterogeneity, preventing water penetration, and maintaining the integrity, rigidity, and fluidity of the plasma membrane (Abe et al., 2009). In both mammals and fungi, cells regulate sterol homeostasis by multiple transcriptional and post-transcriptional feedback mechanisms. In *C. albicans*, ergosterol biosynthesis is mainly regulated by transcription factors, such as Upc2p, Efg1p, and Ndt80p (Sellam et al., 2009; Prasad et al., 2010; Schneider and Morschhauser, 2015). Compared with the extensive research on these transcription factors, little is known about post-transcriptional regulation of sterol homeostasis in *C. albicans*.

In *Saccharomyces cerevisiae*, sterol content can be controlled by insulin-induced genes (INSIGs) interacting directly with proteins containing sterol-sensing domains (SSDs), which is an important post-transcriptional regulation (Espenshade and Hughes, 2007). The Nsg1p and Nsg2p, classified to INSIGs in *S. cerevisiae*, control the sterol-dependent degradation of HMG-CoA reductase. Nsg1p and Nsg2p increase the stabilization of Hmg2p by a direct interaction with the SSDs and decrease the endoplasmic reticulum (ER)-associated degradation (ERAD) of HMG-CoA reductase. This process is dependent on the lanosterol and gernanylgeranyl pyrophosphate (GGPP) levels (Flury et al., 2005). Lanosterol promotes the association of Nsg1p and Hmg2p and improves the stability of Hmg2p. When the content of lanosterol is low and GGPP is high, the physical association between Hmg2p and Nsg1p is compromised. Both Hmg2p and Nsg1p then undergo ubiquitination and degradation by ERAD pathways (Theesfeld and Hampton, 2013). *NSG2* was considered to have a similar function to *NSG1*. The *NSG1*/*NSG2* double deficient *S. cerevisiae* is hypersensitive to lovastatin, which reduces cholesterol levels through a competitive inhibition of HMG-CoA reductase in mammals.

We investigated whether there are INSIG genes regulating the ergosterol biosynthesis in *C. albicans*. Through similarity comparisons, we identified that *C. albicans* C3_02820C_A (*ORF19.273*), containing an INSIG domain, is orthologous to *NSG2* in *S. cerevisiae* and therefore we designated it as *NSG2*. We created a null mutant of *NSG2* in *C. albicans*; in contrast to the functions of *NSG1*/*NSG2* in *S. cerevisiae*, an *NSG2*-deficient *C. albicans* strain improved the sensitivity to azoles and showed no difference to other stresses compared with its parental strain SN152. Hypersensitivity of *nsg2*Δ/Δ to azoles was caused by the imbalance of sterols, especially the increase of eburicol and obtusifoliol. Our study gives a new insight in understanding the synthesis of sterols in *C. albicans* and the roles of sterol homoeostasis in resistance to azole stress.

## Materials and Methods

### Media and Agents

Unless otherwise specified, all the strains were grown in yeast peptone dextrose (YPD, 2% dextrose, 2% peptone, 1% yeast extract) at 30°C. Synthetic complete (SC) medium (2% dextrose, 0.67% yeast nitrogen base, and amino acid mixture) was supplemented with histidine, leucine, or arginine. Fluconazole, ketoconazole, miconazole, lovastatin, terbinafine were purchased from Sigma. DMSO, ether, and cyclohexane were purchased from Sinopharm Chemical Reagent, Co., Ltd.

### Strain Construction

The *C. albicans* strains used in this study are listed in Supplementary Table S1 and all the oligonucleotides used are listed in Supplementary Table S2. In summary, to generate the null *C. albicans NSG2* mutant, the two alleles were sequentially disrupted with the *C. maltosa LEU2* and *C. dubliniensis ARG4* markers. A complementing *NSG2* gene was introduced on a DNA fragment containing the *C. dubliniensis HIS1* marker (Noble and Johnson, 2005). The auxotrophic markers were amplified from plasmid pSN69, pSN52, and pSN40 using the primers of LY232 and LY233 (Schaub et al., 2006). In order to generate the homologous complementing, we designed P1 and P3 primers to amplify the 289 bp cassette at 117 bp upstream of *NSG2.* Primers P4 and P6 were used to amplify the 201 bp cassette at 123 bp downstream of *NSG2.* Fusion PCR was used to amplify the disruption cassette of *NSG2* (NSG2Δ::C.m.Leu2 and NSG2Δ::C.d.ARG4 disruption cassette). These cassettes were transformed into SN152 using the LiAC protocol. The correct *NSG2* disruptions were confirmed by PCR using the identification primers. The same strategy was used to construct the *NSG2* reintroduction strains and the oligonucleotides used are listed in Supplementary Table S2. The reintroductions were identified by selection for the *HIS1* auxotrophic marker.

### Drug Susceptibility Testing

MIC_80_ was determined by growth in RPMI1640 medium for 24 h as mentioned in CLSI M27-A (Raiesi et al., 2017). Briefly, *C. albicans* was cultured in YPD for 16 h, and washed with PBS for three times. The fungal suspension was adjusted to 5 × 10^3^ CFU/ml with RPMI1640 medium. 100 μl of such inoculum was added into the 96 well plates, except for the first well which was the medium control. Drugs were added to the second well and diluted twofold serially. After cultured in 30°C for 24 h, OD_630_ was measured by microplate reader. The lowest concentration of antifungal drug that was sufficient to inhibit 80% of fungal growth was designated as MIC80.

Sensitivities of strains to different drugs were further examined on the YPD agar plates by spot assay. Drugs were dissolved in DMSO as a stock solution and added to the prewarmed YPD media containing 2% agar. *C. albicans* were cultured in YPD for 16 h and adjusted to a density of OD_600_ = 0.40 in PBS buffer. Five suspensions of fivefold serial dilution of *C. albicans* were spotted to plates containing drugs at the indicated concentrations in a volume of 3 μl. Finally, the plates were incubated at 30°C for 2 days.

### Transmission Electron Microscopy

The membrane changes caused by the disruption of *NSG2* or by fluconazole were imaged by transmission electron microscopy. Briefly, 500 μl of *C. albicans* cultured in YPD medium overnight were added to 50 ml fresh YPD medium with or without indicated concentrations of fluconazole and incubated at 30°C for 8 h with shaking at 200 rpm. After centrifugation, cell pellets were fixed with 2.5% glutaraldehyde in 0.1 M cacodylate buffer at 4°C for 24 h. Post-fixation was carried out in 1% osmium tetroxide in cacodylate buffer. After that, the pellets were dehydrated in acetone and embedded in epon. Ultra thin sections were stained with 12.5% alcoholic uranyl acetate and viewed using a HITACHI H-7650 transmission electron microscope at 80 kV. Ultrastructure of SN152 and *NSG2* null mutant were compared to assess the effects of compounds.

### Determination of Cell Membrane Permeability by PI Staining

The concentration of *C. albicans* was adjusted to 5 × 10^6^ CFU/ml with RPMI1640 medium containing different concentrations of fluconazole. A total of 1.5 ml RPMI1640 medium with *C. albicans* were transferred to different tubes and shaken at 200 rpm for another 8 h. Cells were centrifuged to remove the RPMI1640 medium and washed three times with PBS. 1 × 10^6^ cells were stained by 2 μM propidium iodide (PI) for 50 min at 30°C. After staining, cells were washed three times with PBS and fixed with 4% paraformaldehyde. BD flow cytometry was used to detect the percentage of PI positive cells (Novickij et al., 2017).

### Quantitation of the Percentage of Ergosterol

The method of Arthington-Skaggs et al. (1999) with slight modifications was used to determine the effect of *NSG2* on ergosterol biosynthesis in *C. albicans*. Total intracellular ergosterol was quantified in the presence and absence of fluconazole in *C. albicans*. Briefly, 100 μl of different strains were inoculated into 10 ml of YPD with or without the indicated drugs and incubated at 30°C for 8 h at 200 rpm. Cells were washed twice with ddH_2_O, and the weight of wet cell pellet was adjusted to 0.7 g. Further, 3 ml of 15% NaOH resolved in 90% ethanol was added to each pellet and mixed thoroughly. The suspension was incubated at 90°C for 1 h in water bath. Sterols were extracted by addition of 1 ml of distilled water and 3 ml of *n*-heptane. The mixture was vortexed vigorously for 5 min and allowed to stand for 15 min. The heptane layer was transferred to clear glass tubes and stored at -20°C. Prior to analysis 1 ml of each sterol aliquot was diluted fivefold in 100% ethanol and scanned between 230 and 300 nm using UV-visible spectrophotometer. A similar dilution of heptane in 100% ethanol was used as the blank. 50 μg of cholesterol was added to each sample as the internal reference for the quantification of other sterols. The silylation of sterols is derivatized with TMSI in 30 ml pyridine for 1 h at 60°C. A 2 μl aliquot was injected into the gas chromatograph-mass spectrometry (Finnigan Voyager, United States) with HP-50 columns (50% Phenyl-50% methylpolysiloxane, 30 m × 0.25 mm × 0.25 μm). Sterols of interest were identified by their relative retention times and mass spectra compared with the sterol profiles of NIST.

### Systemic Murine Candidiasis Model

Groups of BALB/C female mice (6–8 weeks) were infected with different strains via lateral tail vein with 200 μl saline containing 5 × 10^5^ CFU *C. albicans*. Fluconazole was administered to the infected mice at a dose of 2 mg/kg once a day intraperitoneally for a week starting 2 h after the injection with *C. albicans*. Mice were monitored daily for survival for a period of 50 days. Kaplan–Meier analyses were used to indicate the survival probabilities and Log-rank testing was used to evaluate the significance of survival curves.

## Results

### The Deficiency of *NSG2* Specifically Increased Sensitivity of *C. albicans* to Azoles

**FIGURE 1:**
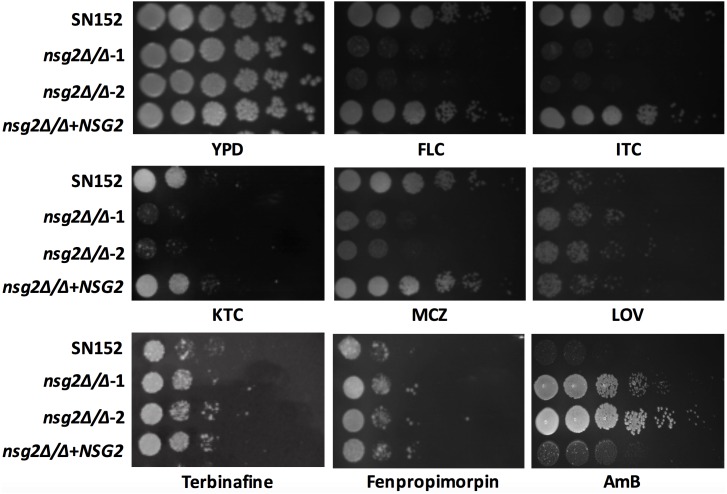
The *nsg2*Δ/Δ strain compromises adaptation to azoles. Spot assays comparing the sensitivity of the wild-type SN152, the *NSG2*-deficient and the revertant strain. Cells were grown in YPD for 16 h, diluted, and spotted onto the plates with the indicated concentrations of different drugs. The strains were cultured on solid YPD medium and photographed after 2 days growth at 30°C. The concentrations of each drug: fluconazole, 5 μg/ml, itraconazole, 2.5 μg/ml, ketoconazole, 2.5 μg/ml, miconazole, 2.5 μg/ml, lovastatin, 8 μg/ml, terbinafine, 2.5 μg/ml, fenpropimorpin, 2.5 μg/ml, amphotericin B, 0.5 μg/ml.

In *S. cerevisiae*, the disruption of both *NSG1* and *NSG2* leads to an increased sensitivity to lovastatin, which inhibits the synthesis of mevalonate. In our study, *C. albicans NSG2* disrupted strains were constructed (Supplementary Figure S1). The growth rate and the formation of hyphae or biofilm were investigated and we found that *NSG2* disruption had no effect on the proliferation and morphological transformation of *C. albicans* (Supplementary Figure S2). Unlike *S. cerevisiae*, in *C. albicans* lovastatin (8 μg/ml) strongly and equally inhibited the growth of both the wild-type SN152 and the *nsg2*Δ/Δ strain (**Figure 1**), which suggested that *NSG2* has distinct functions in *S. cerevisiae* and *C. albicans*. There are three classes of antifungal drugs targeted to the sterol synthetic pathway: allylamines (terbinafine, butenafine) target squalene epoxidase (Erg1p), triazoles (fluconazole, ketoconazole, etc.) target the lanosterol 14α-demethylase (Erg11p) and morpholines (amorolfine, fenpropimorph) target C-8 sterol isomerase (Erg2p) and C-14 sterol reductase (Erg24p). Antifungal susceptibility was determined by spot assays and MIC_80_ (**Figure 1** and **Table 1**). The strains *nsg2*Δ/Δ*-1* and *nsg2*Δ/Δ*-2* were two independent transformants; they behaved almost identically. The deletion of *NSG2* in *C. albicans* significantly enhanced the susceptibility to azoles including fluconazole, itraconazole, miconazole and ketoconazole, but reduced susceptibility to amphotericin B. However, *NSG2* deletion didn’t affect the susceptibility of *C. albicans* to terbinafine, fenpropimorph or lovastatin, which is different from lovastatin-sensitive phenotypes of *nsg2*Δ/Δ in *S. cerevisiae*.

**Table 1:** MIC_80_ (μg/ml) values of azoles for different strains in RPMI1640 following 48 h incubation.

	SN152	nsg2Δ/Δ-1	nsg2Δ/Δ-2	nsg2Δ/Δ+NSG2
Lovastatin	8	8	8	8
Fluconazole	32	8	4	64
Ketoconazole	16	4	4	16
Itraconazole	8	2	2	8
Miconazole	8	2	2	4
Terbinafine	4	4	4	4

### The Depletion of *NSG2* Disrupted the Integrity and Function of the Cell Membrane

**FIGURE 2:**
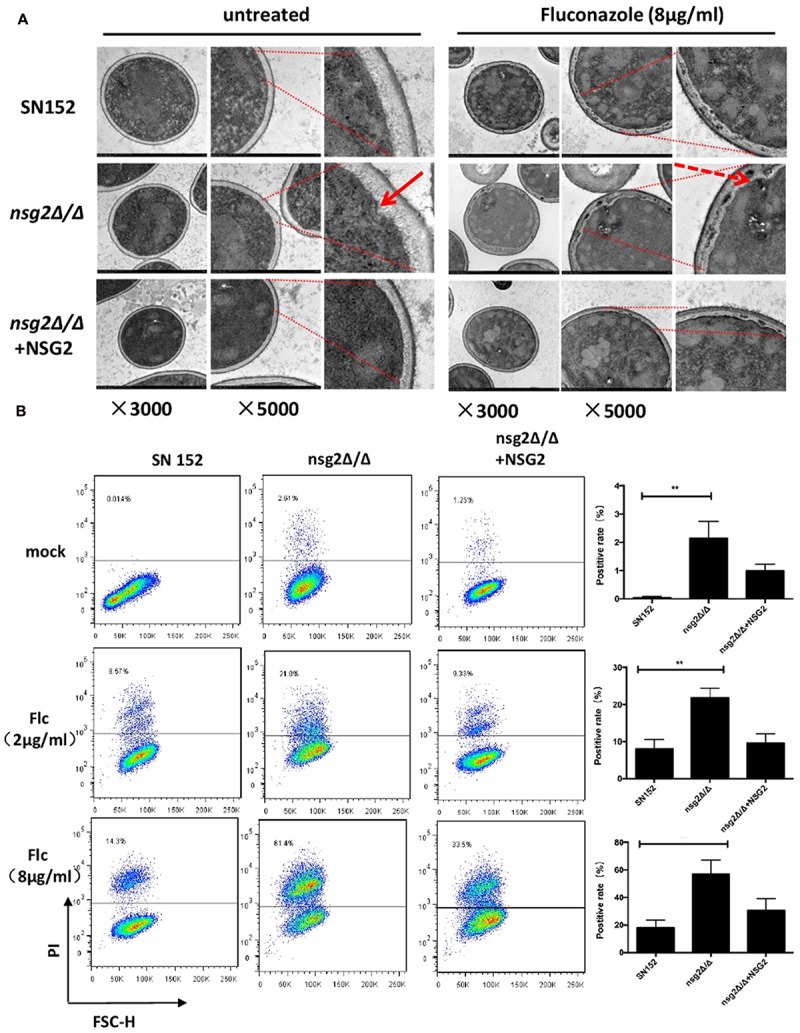
The integrity and functions of cell membrane by *NSG2* disruption. **(A)** Ultra-structure images of *Candida albicans* SN152, *nsg2*Δ/Δ and *nsg2*Δ/Δ+NSG2 strains in the presence or absence of 8 μg/ml of fluconazloe for 16 h. Solid arrow indicated the slight damage in the membrane of *nsg2*Δ/Δ and the dashed arrow indicated the extensive solubilization of the cytoplasmic membrane in the presence of 8 μg/ml of fluconazole. The magnification was indicated at the bottom of each picture. **(B)** Representative flow cytometric profiles and graph showing the proportions of PI^+^-gated cells in SN152, *nsg2*Δ/Δ and *nsg2*Δ/Δ+NSG2 treated with fluconazoles or not. Cells were treated with fluconazoles for 8 h and stained by 2 μM PI for 50 min. ^∗∗^*p* < 0.01 (two-tailed unpaired *t*-test).

Ergosterol plays an important role in cell membrane integrity, permeability, and cell polarization. The ultra-structure of membranes of the SN152 and *nsg2*Δ/Δ strains was monitored with or without the action of fluconazole via transmission electron microscopy. The deletion of *NSG2* resulted in slight damage to the plasma membrane of *C. albicans* (**Figure 2A**). Moreover, in the presence of 8 μg/ml of fluconazole the cell membrane of strain *nsg2*Δ/Δ shriveled dramatically and a large amount of entocyte was leaked into the gap of the cell membrane and the cell wall. Based on this observation, PI staining was used to evaluate the the effect of *NSG2* deletion on cell membrane permeability (**Figure 2B**). In the absence of fluconazole, only a very small proportion of SN152 was stained by PI. However, in *NSG2*-deficient strains, approximately 2% of cells were PI-positive due to the slight cell membrane damage that we observed under the transmission electron microscopy. Consistent with this, the percentage of PI-positive cells in *NSG2* disrupted strains increased significantly with the addition of 2 or 8 μg/ml of fluconazole (**Figure 2B**). These data suggested that the loss of *NSG2* gene led to changes in cell membrane permeability and a slight impairment of cell membrane function. However, when we stained the yeast and cells in the early stage of hyphal formation with filipin, which combines specifically with ergosterol, we found that *nsg2*Δ/Δ stained normally with an ergosterol-rich domain in both the yeast cells and hyphae (Supplementary Figure S3). This suggests that cell membrane damage to the *nsg2*Δ/Δ strains is not caused by ergosterol loss.

### A Deficiency of *NSG2* Increased the Accumulation of the C14-Methylated Sterols

**FIGURE 3:**
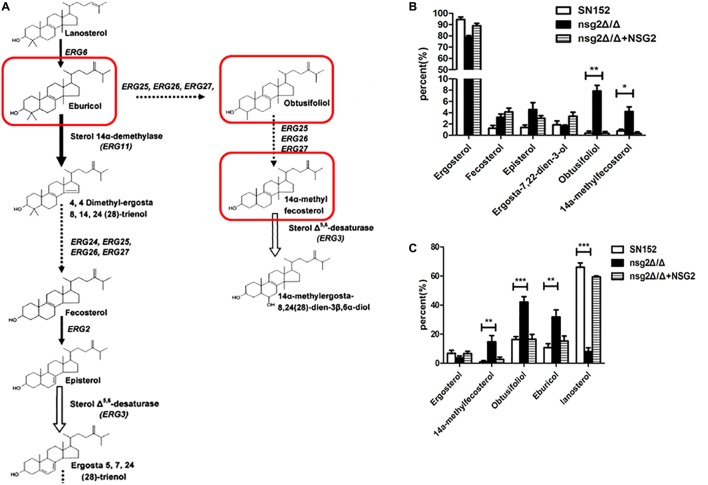
Determination of sterols in *NSG2*-deficient strains by gas chromatography–mass spectrometry (GC–MS). **(A)** Sterol synthesis pathway in *C. albicans*. The red box is on behalf of accumulated sterols in *nsg2*Δ/Δ. Broken arrows, multiple enzymatic steps; solid arrows, single enzymatic step (Martel et al., 2010b). **(B)** Sterol compositions of SN152, *nsg2*Δ/Δ and *nsg2*Δ/Δ+NSG2 which were cultured in YPD medium for 16 h and extracted by NaOH agents. **(C)** Sterol compositions of SN152, *nsg2*Δ/Δ and *nsg2*Δ/Δ+NSG2 which were treated with 8 μg/ml of fluconazole for 6 h. The percentage of each sterol is calculated by all detected sterols in each sample. ^∗^*p* < 0.05, ^∗∗^*p* < 0.01, ^∗∗∗^*p* < 0.001 (two-tailed unpaired *t*-test).

The azoles inhibit fungal growth due to a reduction of ergosterol and an increase in toxic sterols, which leads to decreased fluidity and permeability of the plasma membrane. Therefore, we measured the compositions of sterols in the strains by gas chromatography–mass spectrometry (GC–MS). In the absence of fluconazole, ergosterol was the major sterol in both SN152 and *nsg2*Δ/Δ. But the percentage of obtusifoliol and 14α-methylfecosterol formed upstream of the Erg11p step were higher in *nsg2*Δ/Δ than that in SN152 (**Figures 3A,B**). What’s more, the obtusifoliol and 14α-methylfecosterol accumulated in *nsg2*Δ/Δ strains both contain a 14α-methyl group which is fungistatic for yeast (Vale-Silva et al., 2012; Keller et al., 2015). These data suggested that *NSG2* played an important role in regulating the balance of different sterols. This phenomenon was more obvious in *C. albicans* responding to fluconazole. With the treatment of 8 μg/ml of fluconazole, the percentage of ergosterol in both SN152 and *nsg2*Δ/Δ decreased to about 4% and the percentage of sterols with a C14-methyl group increased. Fluconazole inhibited the activity of lanosterol 14α-demethylase (Erg11p) and induced accumulation of lanosterol in SN152 and the reintegrate strain. However, when *NSG2* was disrupted, the proportion of eburicol, obtusifoliol, and 14α-methylfecosterol was significantly enhanced while the percentage of lanosterol in *nsg2*Δ/Δ was lower than that in SN152 (**Figure 3C**). When using the squalene epoxidase inhibitor terbinafine, the difference of distribution and composition of sterols between SN152 and the *NSG2* null mutant was not significant (Supplementary Figure S4). Finally, we concluded that the specific hypersensitivity of *nsg2*Δ/Δ to azoles was attributed to the slight reduction of ergosterol and the accumulations of sterols with 14α-methyl group. The role of *NSG2* on the content of sterols may be dependent on a direct interaction between Nsg2p and Erg11p, because the transcriptional levels of *ERG1*, *ERG7*, *ERG11*, *ERG2*, and *ERG3* were not changed in the *nsg2*Δ/Δ strain in comparison to those in SN152 (Supplementary Figure S5).

### The Disruption of *NSG2* in *C. albicans* Increased Efficacy of Fluconazole Treatment in a Murine Model of Systemic Candidiasis

**FIGURE 4:**
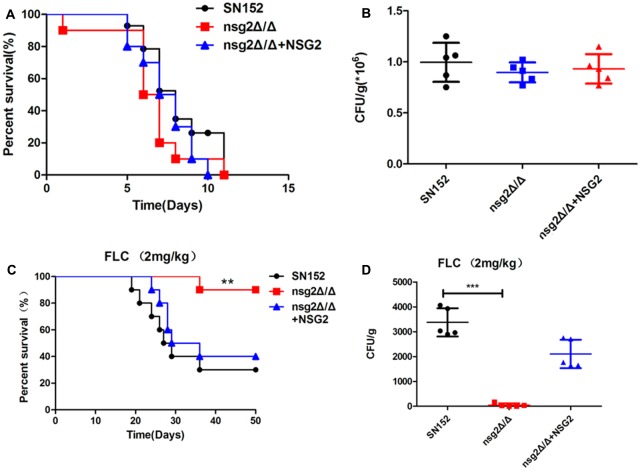
*NSG2*-deficient strains were sensitive to fluconazole *in vivo*. **(A)** Survival curves of BALB/C mice infected by SN152, *nsg2*Δ/Δ or *nsg2*Δ/Δ+NSG2 strains. A total of 3 × 10^5^ CFU cells were injected to female BALB/C (6–8 weeks) through tail vein and observed until all mice dead. **(B)** Kidney CFU assay in mice with systemic candidiasis after infected with 3 × 10^5^ CFU after 2 days. **(C)** Survival curves of BALB/C mice infected by SN152, *nsg2*Δ/Δ or *nsg2*Δ/Δ+NSG2 and treated with 2 mg/kg fluconazole for 1 week, ^∗∗^*p* < 0.01 (Log-rank test). **(D)** Kidney CFU assay in mice with systemic candidiasis after treatment with 2 mg/kg fluconazole for 7 days. ^∗∗∗^*p* < 0.001 (two-tailed unpaired *t*-test).

To investigate the effects of *NSG2* in maintaining the virulence of *C. albicans* in mice, we compared the survival rate and the renal fungal burden of strains in BALB/C mice. Without treatment with fluconazole, the survival rate and the renal fungal burden of the *NSG2* null mutant were similar to those in SN152 and the reintegrate strain (**Figures 4A,B**). We then investigated whether *NSG2* disruption improved the outcome of mice treated with fluconazole *in vivo*. The results showed that the *NSG2* null mutant was almost totally cleared from the kidney. When treated with 2 mg/kg fluconazole, the survival percentage of mice infected with *nsg2*Δ/Δ and was about 90% during 50 days (**Figures 4C,D**). In contrast, only 30–40% of the mice infected with SN152 or the *NSG2* reintroduction strain were alive with the treatment of fluconazole after 50 days. Overall, *NSG2* maintained the *C. albicans* resistance to azoles *in vitro* as well as *in vivo*.

## Discussion

The sterol biosynthesis pathway in eukaryotic cells is conserved and subject to a strict regulatory mechanism (Prasad and Singh, 2013). In *C. albicans* the transcriptional regulation of ERG genes includes primarily the transcriptional factors Upc2p, Efg1p, and Ndt80p (Silver et al., 2004; Sellam et al., 2009; Prasad et al., 2010). Currently, the post-transcriptional regulation of the activity or stability of enzymes in the process of ergosterol synthesis is poorly studied. In mammals and yeast, the synthesis and uptake of sterol is dependent on a strict feedback control mechanism by the membrane-bound transcription factor sterol regulatory element binding protein (SREBP) (Bien and Espenshade, 2010; Raychaudhuri et al., 2012). One of the best studied functions of SREBP is the regulation of HMGR transcription and sterol-dependent degradation, which is dependent on INSIG proteins (Espenshade and Hughes, 2007). Nsg2 in *C. albicans*, which is homologous to the *S. cerevisiae* INSIG protein Nsg2p, was found to be associated with resistance to azoles in our study, as the *C. albicans NSG2* null mutant strain showed hypersensitivity to azoles. *C. albicans* cell membranes appeared to be slightly damaged in the absence of *NSG2* and more seriously damaged in the presence of fluconazole.

Through the GC–MS analysis of the constitutions of sterols, we found that the hypersensitivity to azoles in *nsg2*Δ/Δ was related to the increased eburicol, obtusifoliol, and 14α-methylfecosterol proportions, which were almost absent in the normal strains. Eburicolis is converted to obtusifoliol and then 14α-methylfecosterol in two successive steps under the catalyzation of Erg25p, Erg26p and Erg27p, then to the toxic 14α-methylergosta-8,24 (Maguire et al., 2014)-dien-3β,6α-diol under the action of Erg3p. In our research, the presence of the toxic 14α-methylergosta-8,24 (Maguire et al., 2014)-dien-3β,6α-diol has not been identified in *nsg2*Δ/Δ. Sanglard et al. (2003) have reported the existence of lots of obtusifoliol and a small amount of 14α-methylfecostreol in *erg11*Δ/Δ mutants. This is very similar to the sterol distribution changes caused by *NSG2* disruption in our study, although *NSG2*-defecient strains have a lower proportion of obtusifoliol compared with *erg11*Δ/Δ. In addition, *NSG2*-disrupted mutants showed similar antifungal drug susceptibility compared with the heterozygous *ERG11* mutants in the studies of Sanglard et al. (2003). Both of the two mutants were more sensitive to azoles and more resistant to amphotericin B compared to their wild-type strains. But there is no difference in the susceptibility to terbinafine in *NSG2*-disrupted mutants and the heterozygous *ERG11* mutants. These changed drug sensitivities indicated that the deficiency of *NSG2* may decrease the activity or stability of Erg11p and result in the changed cell membrane properties.

On the other hand, the accumulation of obtusifoliol and 14α-fectosterol could be related to the disfunction of Erg3p which catalyzes the convention of 14α-fectosterol to 14α-methylergosta-8,24 (Maguire et al., 2014)-dien-3β,6α-diol in *C. albicans* treated with azoles. However, *erg3*Δ/Δ is resistant to azoles and sensitive to amphotericin B, which is opposite to the phenotypes of the *nsg2*Δ/Δ. Consistent with this, obtusifoliol was not detected in *erg3*Δ/Δ. It is speculated that *NSG2*-disruption may not affect the function of Erg3p on the consideration of evidence above.

The toxicity of obtusifoliol and its effect on cell membrane properties have not been fully investigated. However, there is evidence that shows the accumulation of obtusifoliol in sterols may result in an increase of *C. albicans* susceptibility to azoles. Specifically, Kelly et al. (1997) found higher obtusifoliol proportion in azole-sensitive clinical strains than that in drug-resistant strains. In previous studies, the mechanism of fluconazole resistance in *C. albicans* biofilms may be associated with the increased obtusifoliol in planktonic cells, which are sensitive to aozles (Mukherjee et al., 2003). In *C. neoformans*, the products formed by *ERG27* inhibition, such as eburicol and obtusifolione, cannot support its growth (Mysiakina and Funtikova, 2007). Our research showed that obtusifoliol and eburicol were accumulated in *nsg2*Δ/Δ treated with fluconazole or not, which may closely be related to its sensitivity to fluconaozle and the membrane disruption. Consistently, *nsg2*Δ/Δ mutants were almost fully cleared from the kidneys of systemically infected mice by treatment with fluconazole *in vivo*. Given the critical roles of sterols in controlling the efficacy of fluconazole, Nsg2p may be a potential target for the elimination of *C. albicans* resistance to azoles.

Recently, Maguire et al. (2014) elaborated that the SREBPs in *S. cerevisiae* (Hms1) and *C. albicans* (Cph2) have dropped the major role in regulating sterol synthesis and instead primarily regulate filamentous growth. The regulation of sterol synthesis is replaced by the transcriptional factor Upc2p (Sellam et al., 2009). As the function of SREBPs is gradually replaced in the evolution of *C. albicans*, we wonder if the INSIG protein function is still conserved. Our result revealed that *NSG2* containing the INSIG domain maintained the balance of ergosterol and 14α-methylated sterols. These results mean that INSIG proteins may have lost their roles in the synthesis of ergosterol. *NSG2* may affect the stability of the Erg11p, as the accumulation of eburicol, obtusifoliol, and 14α-methylfecosterol in *NSG2*-deficient strains. We tested the transcriptional level of *ERG11* gene and there was no difference between SN152 and the *NSG2* deficient strains whether they were treated with fluconazole or not. These results suggest that Nsg2p may enhance the stability or activity of Erg11p through post-translational regulations. What we have known is that the regulation of Erg11p is co-regulated with several other pathways, such as the synthesis of GPI anchors and the iron deprivation pathway (Prasad et al., 2006; Martel et al., 2010b; Hameed et al., 2011; Yadav et al., 2014). If a new protein is found to act in the regulation of stability or activity of enzymes participating in the ergosterol synthesis, it may be a new target of antifungal drugs.

## Ethics Statement

The research was conducted in accordance with the Declaration of Helsinki and with the Guide for Care and Use of Laboratory Animals as adopted and promulgated by the United National Institutes of Health. All mouse experimental procedures were approved by the Institutional Animal Care and Use Committee of the Second Millitary Medical University.

## Author Contributions

Conceived and designed the experiments: LY and Y-YJ. Performed the experiments: Q-ZL and Y-LQ. Analyzed the data: LW and CZ. Wrote the manuscript: Q-ZL, Y-LQ, and CZ.

## Conflict of Interest Statement

The authors declare that the research was conducted in the absence of any commercial or financial relationships that could be construed as a potential conflict of interest.
