# Supplementary material for: NSG2 (ORF19.273) Encoding Protein Controls Sensitivity of Candida albicans to Azoles through Regulating the Synthesis of C14-Methylated Sterols
Source: Front Microbiol. 2018 Feb 20;9:218. doi: 10.3389/fmicb.2018.00218 (PMC5826172; doi:10.3389/fmicb.2018.00218)
Supplement: Supplementary file 1 [file Presentation_1.PDF]

Tab S1. Stains used in this study

Strain	Parental strain	Key Genotype	Reference
SN152	RM1000	*LEU2/leu2,HIS1/his1,arg4/arg4,URA3/ura3 Δ::imm^{434} IRO1/iro1 Δ::imm^{434}*	(Noble and Johnson 2005)
nsg2-1Δ/Δ *nsg2-2Δ/Δ*	SN152	*nsg2 Δ::C.m.LEU2/nsg2 Δ::C.d.HIS1,his1Δ / his1 Δ,arg4Δ/arg4Δ,leu2Δ/leu2Δ,URA3/ura3Δ::imm^{434} IRO1/iro1 Δ::imm^{434}*	This study
nsg2Δ/Δ *+NSG2*	*nsg2-1Δ/Δ*	*nsg2Δ /NSG2, his1 Δ / his1 Δ, arg4 Δ / C.a.ARG4, leu2 Δ / leu2 Δ,URA3/ura3 Δ::imm^{434} IRO1/iro1 Δ::imm^{434}*	This study

Tab S2. Primes used for *NSG2* disruption and the detection of transcriptional levels of ERG genes.

**primer**	**sequence**
P1	TCTCTCTTCCTCGCCTTCCT
P3	cacggcgcgcctagcagcggTTCTTCACAAGGGTTGACTTGA
P4	gtcagcggccgcatccctgcTTGATATCTTGCTGGCATCG
P6	TCCTAAAGTCGAGGAACCAAAA
ICL	TTGGATGAATATCTCGCGTTT
ICR	GAATGCCACTGATGAACGAA
UC	TGGGTAAGAACCCAGGAATG
DC	ACGGGTTTTCAAATGTCGTT
Re-P6	cacggcgcgcctagcagcggTCCTAAAGTCGAGGAACCAAAA

DC2	ACGCACGGATATAAGGAATCAT
ERG1-F	ATGAAAGAGTACGTGGGGCT
ERG1-R	CCACTGGGTTGTTTGTTTGC
ERG7-F	TGGGAAGCTGTTAGACCTAAG
ERG7-R	AGAGCCCTCATAATGACACGT
ERG3-F	TCATAGATGGTTACACTGGCCA
ERG3-R	GGATGGAAAGCATGAGAAGCA
ERG11-F	GAGAACGTGGTGATATTGATCC
ERG11-R	GAACCAAGCAGAAGTAGAAGC
ERG2-F	TCGGTACAGCAATTGGGACT
ERG2-R	TTCGGGAATCAATGCACCAG

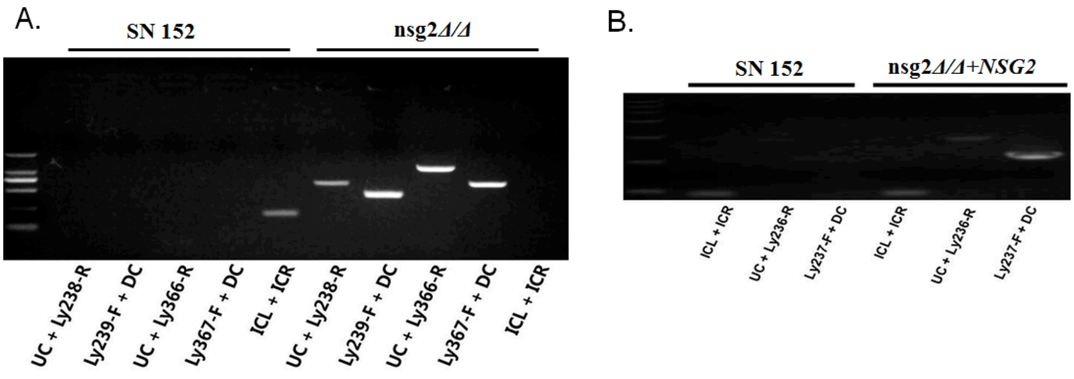

FigS1. Strain construction. A. PCR verification of disruption of *NSG2* by genomic DNA. The oligonucleotides are indicated at the bottom of each line. B. PCR confirmation of the revertant strain by genomic DNA. *NSG2* was reverted to the original sites in the genome.

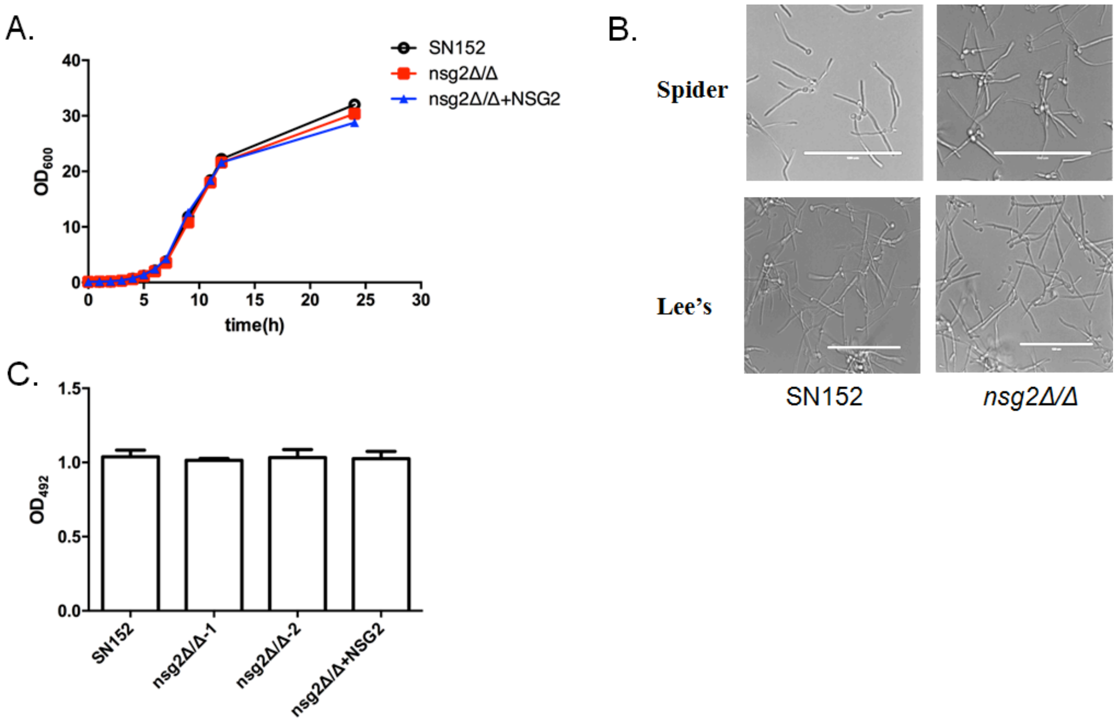

FigS2. *NSG2* has no influence on the proliferation, yeast-hyphae transformation or the formation of biofilm. A. Growth-curves of SN152, *nsg2Δ/Δ*,and the revertant strains in the YPD medium at 30℃ for 24 h. B. Hyphal formation of *C. albicans* SN152 and *nsg2Δ/Δ* cultured in spider and Lee's media at 37℃ for 3 h. C. Biofilms formation measured by XTT staining in different strains.

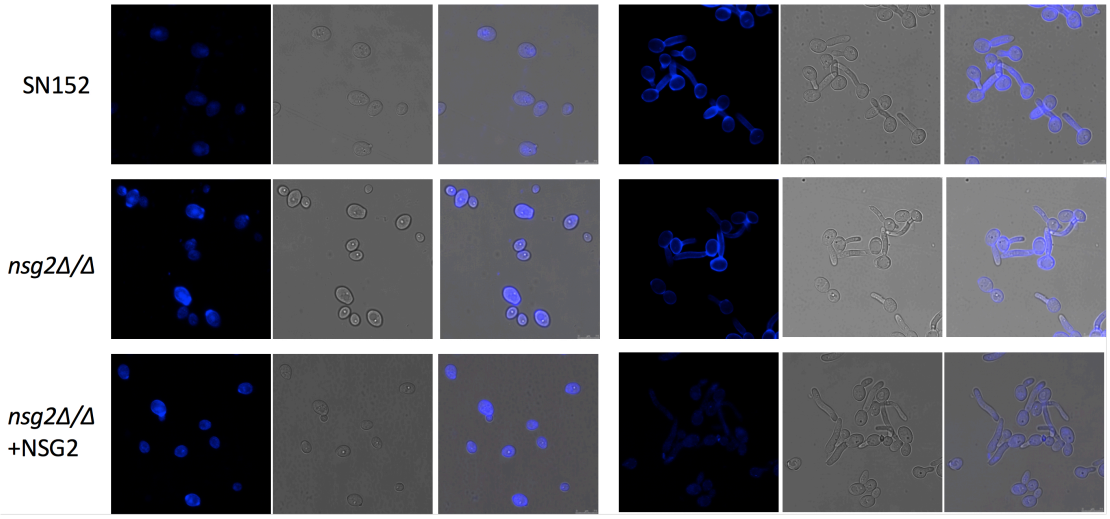

FigS3. Ergosterol stained by filipin on the yeast cells and cells in the early stage of hyphae formation. Strains were cultured in YPD or induced in YNB plus FBS (10%) for 1 h and stained with filipin (10 μg/ml) for 30 min.

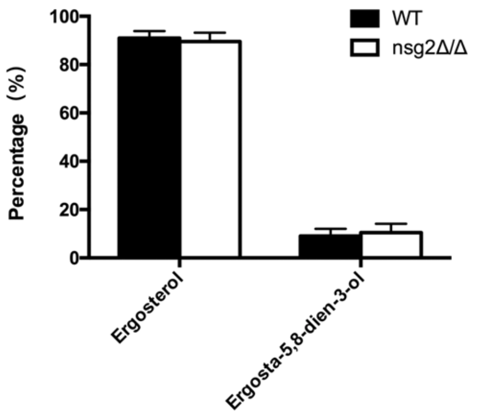

FigS4. Percentage of sterols in the wild tpe (WT) SN152 and *nsg2Δ/Δ* strains by GC-MS in the presence of 4 μg/ml of tebinafine. *C. albicans* SN152, *nsg2Δ/Δ* were treated with 4 μg/ml of tebinafine for 6h, extracted by NaOH agents and analyzed by GC-MS.

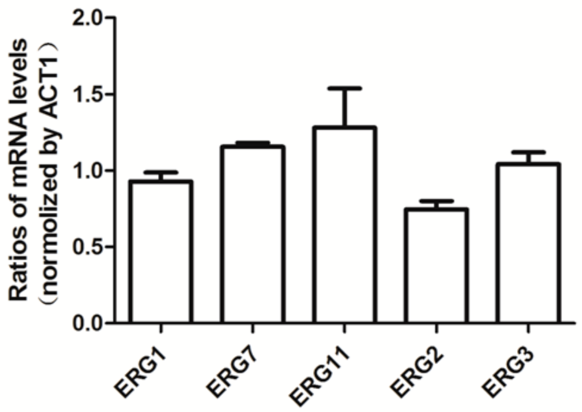

FigS5. Transcriptional level of genes in sterol synthesis pathway determined by q-PCR. SN152 and *nsg2Δ/Δ* were cultured in YPD for 3 h. The expression of ERG genes were normalized by *ACT1* and the ratios were calculated by *nsg2Δ/Δ* versus SN152.
